# Quantification of Visual Field Variability in Glaucoma: Implications for Visual Field Prediction and Modeling

**DOI:** 10.1167/tvst.8.5.25

**Published:** 2019-10-17

**Authors:** Alessandro Rabiolo, Esteban Morales, Abdelmonem A Afifi, Fei Yu, Kouros Nouri-Mahdavi, Joseph Caprioli

**Affiliations:** 1 Stein Eye Institute, David Geffen School of Medicine, University of California Los Angeles, Los Angeles, California, USA; 2 Department of Ophthalmology, University Vita-Salute, IRCCS San Raffaele, Milan, Italy; 3 Department of Biostatistics, Jonathan and Karin Fielding School of Public Health at UCLA, Los Angeles, CA, USA

**Keywords:** perimetry, visual field progression, heteroscedasticity, weighted linear regression, pointwise exponential regression, regression modeling, prediction

## Abstract

**Purpose:**

To quantify visual field (VF) variability as a function of threshold sensitivity and location, and to compare weighted pointwise linear regression (PLR) with unweighted PLR and pointwise exponential regression (PER) for data fit and prediction ability.

**Methods:**

Two datasets were used for this retrospective study. The first was used to characterize and estimate VF variability, and included a total of 4,747 eyes of 3,095 glaucoma patients with six or more VFs and 3 years or more of follow-up. After performing PER for each series, standard deviation of residuals was quantified for each decibel of sensitivity as a measure of variability. A separate dataset was used to test and compare unweighted PLR, weighted PLR, and PER for data fit and prediction, and included 261 eyes of 176 primary open-angle glaucoma patients with 10 or more VFs and 6 years or more of follow-up.

**Results:**

The degree of variability changed as a function of threshold sensitivity with a zenith and a nadir at 33 and 11 dB, respectively. Variability decreased with eccentricity and was higher in the central 10° (*P* < 0.001). Differences among the methods for data fit were negligible. PER was the best model to predict future sensitivity values in the mid term and long term.

**Conclusions:**

VF variability increases with the severity of glaucoma damage and decreases with eccentricity. Weighted linear regression neither improves model fit nor prediction. PER exhibited the best prediction ability, which is likely related to the nonlinear nature of long-term glaucomatous perimetric decay.

**Translational Relevance:**

This study suggests that taking into account heteroscedasticity has no advantage in VF modeling.

## Introduction

Glaucoma is a chronic optic neuropathy characterized by typical modifications of the optic nerve head, retinal nerve fiber layer, and visual field (VF). White-on-white automated perimetry is the standard used for the evaluation and follow-up of patients suffering from glaucoma and correlates with the patients' disability and quality of life.^1^ The timely identification of clinically significant rates of perimetric progression should prompt consideration of treatment escalation to preserve visual function.

The perimetric examination is notoriously disturbed by variability, which confounds the quantification of disease progression. Previous studies have shown that VF variability is not equally spread (homoscedastic) across the entire perimetric range, but varies as a function of threshold sensitivity (heteroscedastic).^2^–^5^ Ordinary least square regression (OLSR) of global indices, VF clusters, or single locations are statistical tools commonly used to assess and quantify glaucoma progression, but they incorrectly assume data homoscedasticity. Heteroscedasticity does not invalidate results obtained with OLSR, but in its presence, this model may not be the best linear unbiased estimator, whereas other linear and nonlinear models may be preferable.^6^ We have previously shown that pointwise exponential regression (PER) can better predict future changes than pointwise OLSR, and the vulnerability of OLSR to data heteroscedasticity could represent one plausible explanation.^7^–^9^

If the degree of heteroscedasticity is known, it can be used to generate a weighted linear model, which assigns higher or lower weight to observations whether they have a lower or higher variance, respectively. However, performance of weighted linear regression has not been reported for VF modeling, and it could be better than classical models because it accounts for heteroscedasticity.

In this study, we characterize variability as a function of threshold sensitivity and test location, and compare weighted pointwise linear regression (PLR) with unweighted PLR and PER in terms of both data fit and prediction ability.

## Methods

This was a retrospective study based on a large cohort of patients with glaucoma treated at the Glaucoma Division of the Stein Eye Institute, University of California, Los Angeles (UCLA). The study was approved by the UCLA Human Research Protection Program, was performed in accordance with the tenets set forth in the Declaration of Helsinki, and complied with the Health Insurance Portability and Accountability Act regulations.

The study was divided into two parts with separate datasets used for each one. The first was to characterize VF variability and estimate the amount of variability for each decibel of threshold sensitivity to develop a weighted linear model. Inclusion criteria for eyes in this part of the study were as follows: diagnosis of any type of glaucoma, six or more reliable VF examinations, and 3 years or more of follow-up. All the tests were carried out with the Humphrey Visual Field perimeter (Carl Zeiss Ophthalmic Systems, Inc., Dublin, CA) with a 24-2 or 30-2, size III white stimulus, and Swedish Interactive Threshold Algorithm (SITA) standard strategy. For the 30-2 examinations, only those locations corresponding to the 24-2 grid were included from the analysis. Reliability criteria included 20% or fewer false positives, 25% or fewer false negatives, and no limitation for fixation losses.^10^

The second part of the study aimed to account for data heteroscedasticity with a weighted PLR, and to compare its performance against unweighted PLR and PER. For this purpose, we used a different dataset of patients with the following criteria: diagnosis of primary open-angle glaucoma (POAG), 10 or more reliable VF examinations, and 6 years or more of follow-up. This second dataset was thus used as a test dataset. All calculations were performed with R statistical software.^11^

The baseline MD and the MD rate of change, obtained by regressing the MD values of each VF series over time, were chosen to be similar between the two groups, and differences were assessed with the Mann-Whitney *U* test.

### Analysis of VF Variability

The estimation of VF variability was performed with a method similar to that reported by Russell et al.^2^ Three different regression models (exponential, linear, logistic) were employed to estimate variability. For each VF series, pointwise regressions were calculated on the threshold sensitivities (dB) over time, excluding the two locations corresponding to the blind spot. For the exponential model, the linear trend (negative or positive) was first determined with simple linear regression, and one of two models (decreasing or increasing) was used. The exponential model was based on a logarithm-transformed linear model and was mathematically defined by the following equations:

(\def\upalpha{\unicode[Times]{x3B1}}\def\upbeta{\unicode[Times]{x3B2}}\def\upgamma{\unicode[Times]{x3B3}}\def\updelta{\unicode[Times]{x3B4}}\def\upvarepsilon{\unicode[Times]{x3B5}}\def\upzeta{\unicode[Times]{x3B6}}\def\upeta{\unicode[Times]{x3B7}}\def\uptheta{\unicode[Times]{x3B8}}\def\upiota{\unicode[Times]{x3B9}}\def\upkappa{\unicode[Times]{x3BA}}\def\uplambda{\unicode[Times]{x3BB}}\def\upmu{\unicode[Times]{x3BC}}\def\upnu{\unicode[Times]{x3BD}}\def\upxi{\unicode[Times]{x3BE}}\def\upomicron{\unicode[Times]{x3BF}}\def\uppi{\unicode[Times]{x3C0}}\def\uprho{\unicode[Times]{x3C1}}\def\upsigma{\unicode[Times]{x3C3}}\def\uptau{\unicode[Times]{x3C4}}\def\upupsilon{\unicode[Times]{x3C5}}\def\upphi{\unicode[Times]{x3C6}}\def\upchi{\unicode[Times]{x3C7}}\def\uppsy{\unicode[Times]{x3C8}}\def\upomega{\unicode[Times]{x3C9}}\def\bialpha{\boldsymbol{\alpha}}\def\bibeta{\boldsymbol{\beta}}\def\bigamma{\boldsymbol{\gamma}}\def\bidelta{\boldsymbol{\delta}}\def\bivarepsilon{\boldsymbol{\varepsilon}}\def\bizeta{\boldsymbol{\zeta}}\def\bieta{\boldsymbol{\eta}}\def\bitheta{\boldsymbol{\theta}}\def\biiota{\boldsymbol{\iota}}\def\bikappa{\boldsymbol{\kappa}}\def\bilambda{\boldsymbol{\lambda}}\def\bimu{\boldsymbol{\mu}}\def\binu{\boldsymbol{\nu}}\def\bixi{\boldsymbol{\xi}}\def\biomicron{\boldsymbol{\micron}}\def\bipi{\boldsymbol{\pi}}\def\birho{\boldsymbol{\rho}}\def\bisigma{\boldsymbol{\sigma}}\def\bitau{\boldsymbol{\tau}}\def\biupsilon{\boldsymbol{\upsilon}}\def\biphi{\boldsymbol{\phi}}\def\bichi{\boldsymbol{\chi}}\def\bipsy{\boldsymbol{\psy}}\def\biomega{\boldsymbol{\omega}}\def\bupalpha{\bf{\alpha}}\def\bupbeta{\bf{\beta}}\def\bupgamma{\bf{\gamma}}\def\bupdelta{\bf{\delta}}\def\bupvarepsilon{\bf{\varepsilon}}\def\bupzeta{\bf{\zeta}}\def\bupeta{\bf{\eta}}\def\buptheta{\bf{\theta}}\def\bupiota{\bf{\iota}}\def\bupkappa{\bf{\kappa}}\def\buplambda{\bf{\lambda}}\def\bupmu{\bf{\mu}}\def\bupnu{\bf{\nu}}\def\bupxi{\bf{\xi}}\def\bupomicron{\bf{\micron}}\def\buppi{\bf{\pi}}\def\buprho{\bf{\rho}}\def\bupsigma{\bf{\sigma}}\def\buptau{\bf{\tau}}\def\bupupsilon{\bf{\upsilon}}\def\bupphi{\bf{\phi}}\def\bupchi{\bf{\chi}}\def\buppsy{\bf{\psy}}\def\bupomega{\bf{\omega}}\def\bGamma{\bf{\Gamma}}\def\bDelta{\bf{\Delta}}\def\bTheta{\bf{\Theta}}\def\bLambda{\bf{\Lambda}}\def\bXi{\bf{\Xi}}\def\bPi{\bf{\Pi}}\def\bSigma{\bf{\Sigma}}\def\bPhi{\bf{\Phi}}\def\bPsi{\bf{\Psi}}\def\bOmega{\bf{\Omega}}\begin{equation}{\rm{Decay\ model}}\mbox{:}\quad \ln\left( y \right) = \alpha + \beta \cdot x + \varepsilon ;\end{equation}

$${equation}{\rm{Improvement\ model}}\mbox{:}\quad\ln\left( {Y - y} \right) = \alpha + \beta \cdot x + \varepsilon ,\end{equation}$$

where the dependent variable *y* is the observed threshold sensitivity (dB), *x* the time (years), α the intercept, β the slope of the regression line, ε the random error, and Y equal to normal age-matched and location-matched sensitivity + 2 standard deviations (SDs).^12^,^13^

The OLSR was expressed by the following formula:

$${equation}y = \alpha + \beta \cdot x + {\rm{\varepsilon }}{\rm{.}}\end{equation}$$

A floor of 0 dB was set to prevent negative predicted values.

The pointwise logistic regression was mathematically expressed as:

$${equation}y = {{\rm{\zeta }} \over {1 + {e^{\alpha + \beta \cdot x + {\rm{\varepsilon }}}}}},\end{equation}$$

where α, β, and ζ were model parameters to be estimated.

The residuals, which represent the difference between the predicted and observed values, were used as a measure of variability. The residuals were estimated with the least squares method for exponential and linear regressions, and with the Newton-Raphson method for logistic regression. Analyses were performed with all the residuals pooled together and binned for observed threshold sensitivity, location, and both threshold sensitivity and location. All the data presented in this study were based on the results from the exponential regression, but analyses were repeated using OLSR and logistic regression to estimate the variability and results are reported in the supplementary material. The SDs of the residuals were calculated for each value of threshold sensitivity, and the relationship between these two variables was mathematically summarized. Because it has been shown that such a relationship is nonlinear,^2^ we fitted an exponential function, which is expressed by the following logarithm-transformed linear model:

$${equation}{\rm{ln}}\left( {{\rm{SD}}} \right) = \alpha + \beta \cdot {\rm{Sensitivity,}}\end{equation}$$

where SD is the standard deviation of the residuals, Sensitivity is the threshold sensitivity value (dB), and α and β are, respectively, the intercept and the slope to be estimated from the data. Because the relationship between variability and sensitivity was not monotonic, we also fitted a spline function, which is a piecewise polynomial divided into segments by endpoints called knots. The function is expressed by the following formula:

$${equation}\ln({\rm{SD}})\left\{ \matrix{ \alpha + {\beta_1} \cdot {\rm{Sensitivity}},\ {\rm{for\ Sensitivity}} \le {k_1} \hfill \cr \alpha + {\beta_1} \cdot {\rm{Sensitivity}} + {\beta_2} \cdot ({\rm{Sensitivity}} - {k_1}),\ {\rm{for\ }}{k_1} \lt {\rm{Sensitivity}} \le {k_2} \cdots ,\hfill \cr \alpha + {\beta_1} \cdot {\rm{Sensitivity}} + {\beta_2} \cdot ({\rm{Sensitivity}} - {k_1}) \cdots {\beta_i} \cdot ({\rm{Sensitivity}} - {k_{i - 1}}),{\rm{for}} \lt {\rm{Sensitivity}} \gt {k_{i - 1}} \hfill } \right.\end{equation}$$

where Sensitivity is the integer value of threshold sensitivity ranging from 0 to 35 dB; *k*_1_, *k*_2_, and *k_i_*_–1_ are the sensitivity values corresponding to the first, second, and *i*th − 1 knots, respectively; $(\alpha$ is the intercept to estimate; and β_1_, β_2_, and β*_i_* are the slopes to be estimated from the data for the first, second, and *i*th piece of regression, respectively.

### VF Modeling

Regression analysis was performed at each location on threshold sensitivities over time, excluding test locations corresponding to the blind spot. Locations with a sensitivity of 0 dB in two of three first examinations were excluded from the analysis. The regression models performed were unweighted linear, exponential, and weighted linear.

The simple linear model was defined as follows:

$${equation}{y_{\left[ i \right]}} = \alpha + \beta \cdot {x_{\left[ i \right]}},\end{equation}$$

where *y*_[_*_i_*_]_ is the *i*th known value of observed threshold sensitivity (dB), and *x*_[_*_i_*_]_ is the *i*th known value of the follow-up (years). The unweighted linear (and exponential) models assume that the variance of *y* (and ln[*y*]) are constant for all the observations. The weighted linear regression model has the same specifications of unweighted OLSR, except for the assumption of constant variance. Instead of assuming equal variance, we assume that the *i*th observation, *y*_[_*_i_*_]_, has variance $({v_i}$. We then obtain the weighted regression estimates using a weight

$${equation}{w_{\left[ i \right]}} = {1 \over {{v_i}}}\end{equation}$$

for the *i*th observation.^6^ For the exponential model, the linear trend (negative or positive) was first determined with simple linear regression, and one of two models (decreasing or increasing) was used. The exponential model was based on a logarithm-transformed linear model and was mathematically defined by the following equations:

$${equation}{\rm{Decay\ model}}\mbox{:}\quad \ln({y_{\left[ i \right]}}) = \alpha + \beta \cdot {x_{\left[ i \right]}};\end{equation}$$

$${equation}{\rm{Improvement\ model}}\mbox{:}\quad \ln \left( {Y\left[ i \right] - {y_{\left[ i \right]}}} \right) = \alpha + \beta \cdot {x_{\left[ i \right]}},\end{equation}$$

where *Y*[*i*] is equal to normal age-matched and location-matched sensitivity + 2 SD.^12^,^13^

Because the dynamic range of the instrument is limited, predicted values with linear models that were negative or abnormally high (i.e., over the normal age-matched and location-matched sensitivity + 2 SD) were censored at 0 dB and at the normal age-matched and location-matched sensitivity + 2 SD dB, respectively.^12^,^13^ The exponential model asymptotically tends toward the floor or ceiling, respectively, and does not require censoring.

### Model Evaluation

Models were compared for data fit and prediction ability with the average root-mean-square error (RMSE) as a criterion. For the data fit, all the observations were regressed over the entire follow-up. For prediction ability, regressions were based only on initial observations as defined next, and were used to predict the value of a future observation. To predict VF number 10, we sequentially regressed the first five to the first nine VFs, adding one VF at every iteration (VF_1–5_, VF_1–6_, VF_1–7_, VF_1–8_, and VF_1–9_). To predict VF number 15, we sequentially performed a regression of first five to the first 13 VFs adding two VFs at every iteration (VF_1–5_, VF_1–7_, VF_1–9_, VF_1–11_, and VF_1–13_). To predict VF number 20, we sequentially performed a regression of first five to the first 17 VFs adding three VFs at every iteration (VF_1–5_, VF_1–8_, VF_1–11_, VF_1–14_, and VF_1–17_).

## Results

### Variability Estimation

**Figure 1:**
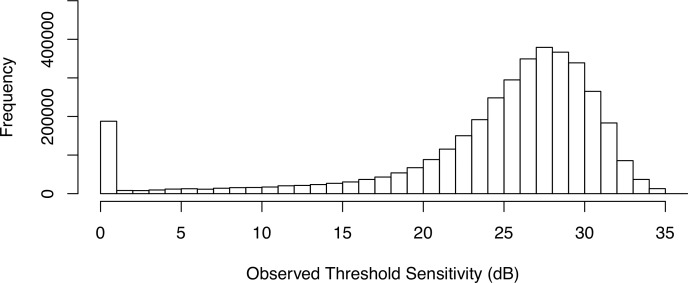
Frequency distribution of the observed sensitivity values.

The baseline characteristics of patients used to estimate variability are reported in Table 1. A total of 4747 eyes of 3095 patients were included in the study. A total of 2,630,628 single observations from 50,589 examinations had a mean (SD) sensitivity of 23.5 (8.3) dB. The frequency distribution of the observed sensitivities is illustrated in Figure 1.

**Table 1:** Characteristics of the Study Population to Estimate Visual Field Variability

Variable	Value
*N* patients/eyes	3095/4747
Age at baseline, y, median (IQR)	66.4 (57.3–73.5)
Follow-up, y, median (IQR)	9.5 (6.5–12.8)
Total number of VFs	50,589
*N* of VF per eye, median (IQR)	9 (7–13)
Baseline MD, dB, median (IQR)	−3.2 (−6.6 to −1.7)
MD rate of change, dB/year, median (IQR)	−0.14 (−0.40 to 0.01)

**Figure 2:**
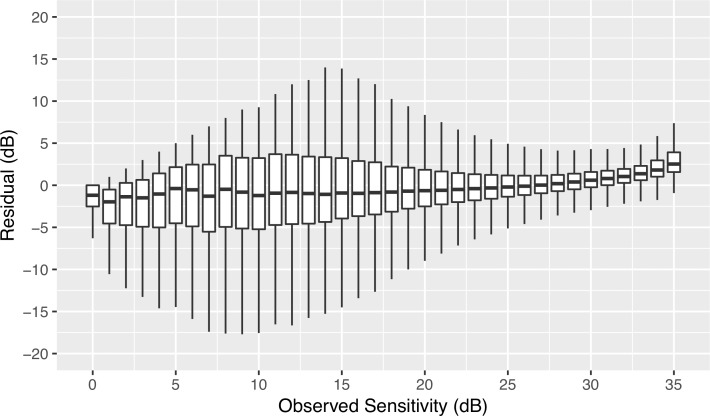
Boxplot of the residual distribution as a function of the values of observed sensitivity.

**Figure 3:**
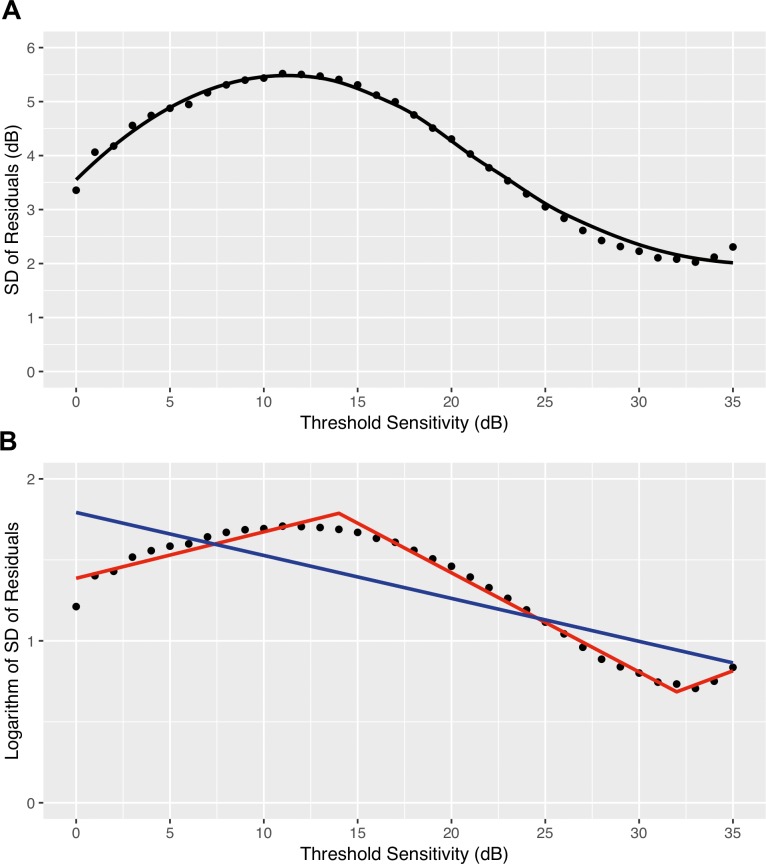
(A) LOESS curve fitting the SD of the residuals, which represents the amount of variability, as a function of the observed threshold sensitivity values. (B) Straight (*blue line*) and spline (*red line*) curves fitting the logarithm of the SD of the residuals as function of the observed threshold sensitivity values. Spline curve has got two knots at 32 and 14 dB.

The distribution of the residuals varied according to the sensitivity level, as shown in Figure 2. The magnitude of variability decreased as threshold sensitivity values increased. The residuals were symmetrically spread around the median down to a sensitivity of approximately 10 dB, then became asymmetric because the dynamic range of the instrument is limited by a floor at 0 dB, which restricts the lower range of predicted values. A similar distribution was seen also when residuals were estimated with OLSR or logistic regression (Supplementary Fig. S1). The SD of the residuals as a function of the observed threshold sensitivity values is shown in Figure 3A, and the value of the SD, which represents the degree of variability for each decibel, is provided in Supplementary Table S1. The variability was minimum at 33 dB (SD of the residuals = 2.0 dB), steadily increased with decreasing sensitivity, peaked at 11 dB (SD of the residuals = 5.5 dB), then dropped at 0 dB to 3.4 dB. As shown in Figure 3B, the relationship between variability and threshold sensitivity was expressed by the following formula: $(\ln \left( {{\rm{SD}}} \right) = - 0.027 \cdot {\rm{Sensitivity}} + 1.79$. Because of this relationship it is better described by a spline curve with two knots at 14 and 32 dB, as expressed by the following formula (Fig 3B):

$${equation}\ln ({\rm{SD}})\left\{ \matrix{ 1.39 + 0.029 \cdot {\rm{Sensitivity}},\ {\rm{for\ Sensitivity}} \le 14{\rm{dB}} \hfill \cr1.39 + 0.029 \cdot {\rm{Sensitivity}} - 0.090 \cdot ({\rm{Sensitivity}} - 14),\ {\text{for\ Sensitivity \ 15\mbox{--}32 dB}.} \hfill\cr 1.53 + 0.029 \cdot {\rm{Sensitivity}} - 0.090 \cdot \left( {{\rm{Sensitivity}} - 14} \right) + 0.104 \cdot ({\rm{Sensitivity}} - 32), \ {\text{for \ Sensitivity}} \gt \text{32 dB} \hfill } \right.\end{equation}$$

As illustrated by the β coefficients of the above equation, the three regression pieces of the spline model have different behaviors. Starting from the 0 dB, the first segment (0–14 dB) had a positive trend with significant variability increase (*P* < 0.0001), the intermediate segment (14–32 dB) had a negative slope with a significant variability reduction (*P* < 0.0001), while the last segment (32–35 dB) had a positive slope with a variability increase (*P* < 0.0001).

The variability curve as a function of the threshold sensitivity showed a similar shape when residuals were estimated with logistic and linear models, as shown in Supplementary Figure S2. With these two models, the variability reached a plateau below 10 dB instead of decreasing as with the exponential model. As illustrated in Supplementary Figure S3, the first piece of the spline (0–14 dB) was almost flat with any significant trend for linear model (*P* = 0.40), while it slightly increased with the logistic model (*P* = 0.004).

**Figure 4:**
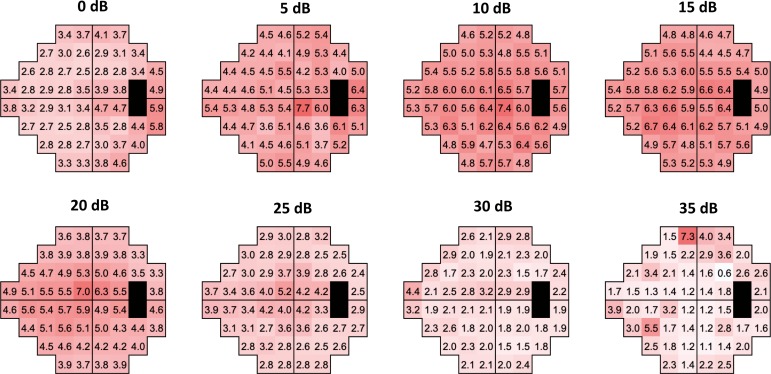
Pointwise SD of the residuals grouped according to the observed sensitivity values. Increased *pink* intensity indicates higher variability.

**Figure 5:**
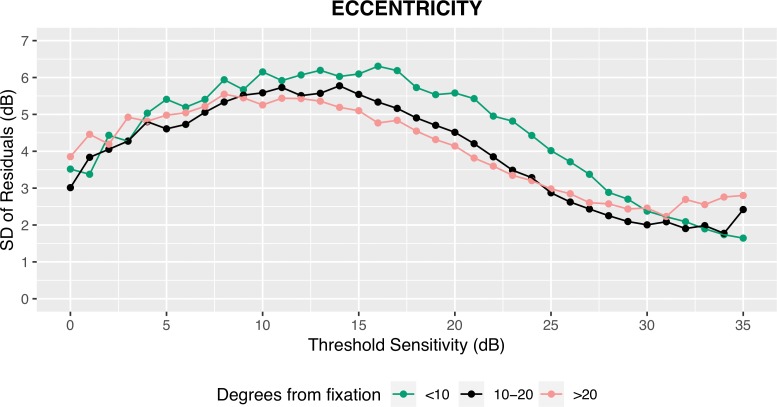
SD of the residuals as a function of the observed threshold sensitivity stratified for degrees from fixation.

Figure 4 shows the values of SD according to the value of threshold sensitivity for each test location. Variability increased with the reduction of the threshold sensitivity, while it decreased with eccentricity. Similar results were obtained when locations were grouped on the basis of their distance from fixation (Fig. 5), or when the analysis was conducted on total deviation values (data not shown). Overall, the variability was larger in the central 10° compared with more than 20° and 10° to 20° areas (*P* < 0.001); conversely, no difference in variability was found outside the central 10° (*P* = 0.61).

### Model Comparisons

The baseline characteristics of patients for this portion of the study are given in Table 2. A total of 261 eyes of 176 patients with POAG were included in the study, with a median follow-up of 15 years and 20 VFs. This cohort of patients did not significantly differ from the previous one with respect to baseline mean deviation (MD; *P* = 0.17) or MD rate of change (*P* = 0.29).

**Table 2:** Main Clinical Data of the Population Used to Compare the Different Models

Variable	
*N* patients/eyes	176/261
Age at baseline, y, median (IQR)	63.6 (55.3–70.4)
Follow-up, y, median (IQR)	15.3 (12.2–17.6)
Number of VFs, median (IQR)	20 (15–25)
Baseline MD, dB, median (IQR)	−3.8 (−8.6 to −1.8)
MD rate of change, dB/y, median (IQR)	−0.16 (−0.38 to −0.04)

IOP, intraocular pressure; VFI, visual field index

**Figure 6:**
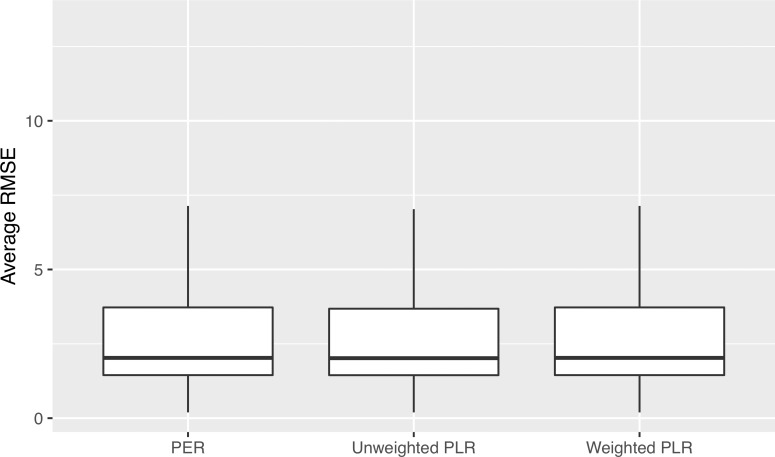
Comparison of model fit for PER, unweighted PLR, and weighted PLR.

For the model fit (Fig. 6), the median (interquartile range [IQR]) RMSE was 2.03 (1.45–3.68) for the unweighted PLR, 2.03 (1.45–3.72) for the weighted PLR, and 2.03 (1.45–3.74) for the PER. The differences among the methods were negligible.

**Figure 7:**
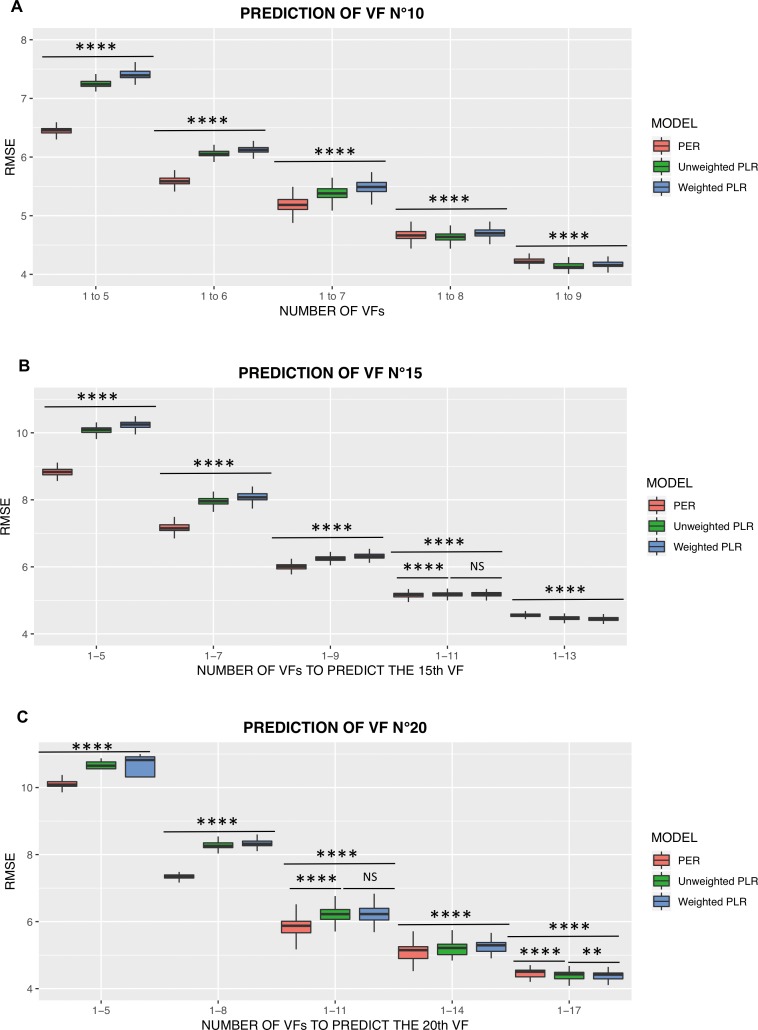
Comparison of ability to predict VF n°10 (A), n°15 (B), and n°20 (C) for PER, unweighted PLR, and weighted PLR. **Significant at *P* < 0.01; ****Significant at *P* < 0.0001; NS: not significant.

Figure 7 shows the RMSEs of the three models to predict VFs numbers 10, 15, and 20. Absolute RMSE values for each method, as well as RMSE differences among methods, became smaller with an increased number of VFs used to make the prediction, and a similar trend was seen for the prediction of the VFs numbers 10, 15, and 20.

To predict VF n°10 (Fig. 7A), PER had the smallest RMSE (*P* < 0.0001) for VF_1–5_, VF_1–6_, and VF_1–7_, while unweighted PLR had the smallest RMSE (*P* < 0.0001) for VF_1–8_, and VF_1–9_. Weighted linear regression had a higher RMSE (*P* < 0.0001) than unweighted PLR for every comparison.

To predict VF n°15 (Fig. 7B), PER had the smallest RMSE (*P* < 0.0001) for VF_1–5_, VF_1–7_, VF_1–9_, and VF_1–11_, while unweighted PLR had the smallest RMSE (*P* < 0.0001) for VF_1–13_. Once again, weighted linear regression had a lower prediction ability (*P* < 0.0001) than unweighted PLR at every comparison, except for VF_1-11_ (*P* = 0.99).

To predict VF n°20 (Fig. 7C), PER had the smallest RMSE (*P* < 0.0001) for VF_1–5_, VF_1–8_, VF_1–11_, and VF_1–14_, while unweighted PLR had the smallest RMSE (*P* < 0.0001 versus PER and *P* = 0.007 versus weighted PLR) for VF_1–17_. Weighted linear regression had a lower prediction ability (*P* < 0.05) than unweighted PLR for every comparison, except compared with unweighted PLR for VF_1–11_ (*P* = 0.35).

Results remained unchanged both for data fit and prediction also when weights were estimated with OLSR and logistic regression (Supplementary Figs. S4 and S5).

## Discussion

In this study, we measured VF variability as a function of threshold sensitivity and test location. We confirmed that variability is not equally spread across the perimetric dynamic range because it varied with threshold sensitivity and it decreases with eccentricity. Specifically, the variability was low in the high-sensitivity range, steadily increased with the worsening of glaucomatous damage with a peak at approximately 10 dB, and then decreased. Based on the values of variability obtained, we modeled weighted PLR on a cohort of POAG patients and tested it against unweighted PLR and PER. Although statistically significant, differences among the methods for data fit were too small to be clinically relevant. Weighted linear regression was not better than unweighted PLR with regard to both model fit and prediction ability. PER was the best model to predict future sensitivity values over the medium and long term, while unweighted PLR was the best model to predict the immediate one or two following VFs. The models' prediction abilities tended to converge with the increase in the number of VFs used to make a prediction.

The early identification of VF progression is one of the most challenging tasks for the glaucoma specialist, and has been the primary outcome of major glaucoma trials.^14^–^17^ Beyond the dichotomy between progression and nonprogression, the quantification of the rate of progression has added importance.^18^ Even healthy patients experience a physiologic decay due to senescence or cataract, but at slow rates.^13^,^19^ Also, glaucoma patients may progress at different speeds, and it is important to distinguish patients with slow rates of decay from those with high rates of decay, because the latter may require more intense follow-up and aggressive therapeutic intervention.^18^ The identification and quantification of VF progression, however, is hampered by the intrinsic variability of the perimetric examination. Several factors have been associated with VF fluctuation, including patient and technician experience, patient motivation, fatigue, uncorrected refractive error, ethnicity, cognitive level, percentage of false-positive and false-negative test responses, time of the day, and season.^10^,^20^–^24^ In addition, the stage of glaucoma is linked to variability.^25^

Variability is an inherent property of VF examination, because the physiologic response to the luminous stimulus relies on a probabilistic concept. The relationship between the probability to see the stimulus and the light brightness is described by the frequency-of-seeing (FOS) curve, and the value of threshold sensitivity indicates the intensity of a stimulus to be able to elicit a response with a probability of 50%.^26^ Previous studies have investigated the relationship between threshold sensitivity and variability with various methods. Old studies defined the variability as the fluctuation of the variance of the threshold sensitivity values in glaucoma patients clinically judged as stable.^27^–^29^ Zulauf and colleagues^27^ evaluated 29 stable glaucoma patients, and found a mean fluctuation of 4.25 dB^2^ over the entire VF with a significant association with threshold sensitivity. Werner et al.^28^ measured variability in 67 glaucoma patients, and confirmed a correlation between variability and threshold sensitivity. Boeglin et al.^29^ quantified the relationship between variability and sensitivity, and found that variability was lowest at 32 dB, then peaked at 10 dB, and decreased below 10 dB. This methodology, however, has limitations because it is impossible to know if a series is truly stable and changes considered fluctuation may instead be actual VF deterioration.

The FOS curve is another way to estimate variability, and is generated with constant stimuli to test a single location with many stimuli whose intensity brackets the estimated threshold sensitivity.^3^,^30^,^31^ In a large cohort of patients, Chauhan et al.^30^ found a correlation between the slope of FOS curve, which is a measure of variability, and threshold sensitivity in healthy subjects, glaucoma suspects, and glaucoma patients. Henson et al.^3^ studied the FOS curves in a heterogeneous cohort of patients, which included 23 healthy subjects and 25 POAG subjects, and confirmed that variability is inversely related to the threshold sensitivity. These findings were further confirmed by Spry and colleagues.^31^ Nevertheless, FOS studies have several limitations because they are conducted on selected “research” subjects whose variability could be lower than the general glaucoma population, are costly, time-consuming, and suffer from a small sample size.

Several studies have measured VF variability with a test–retest approach.^4^,^32^–^34^ With this method, a group of patients is repeatedly tested in a short period of time with the assumption that glaucoma does not progress over a short time period and all the differences in sensitivity values represent variability.^4^ Artes and colleagues^4^ confirmed that variability increases with the severity of glaucoma damage until 10 dB and then decreases. Most of the limitations of FOS studies are also present in test–retest studies. In addition, the latter approach dismisses the possibility of a learning effect, which can be prolonged in some patients.^35^

Recently, Russel et al.^2^ proposed a method based on linear regression of retrospective large-scale longitudinal data from a clinical practice. This approach has several advantages, including the possibility to study very large cohorts of patients tested in a clinical setting, which are highly representative of the general glaucoma population. In our study, we applied a similar method to that described by Russell et al.,^2^ and we obtained similar results both in terms of shape and absolute values of the variability curve.

Previous studies summarized the relationship between threshold sensitivity and standard deviation of the variability. Henson et al.^3^ reported that the variability increased with the reduction in threshold sensitivity as follows: ln(SD) = −0.081 ⋅ Sensitivity (dB) + 3.27, and this formula has been widely used to generate noise in the field of computer-simulated longitudinal VF data.^36^–^38^ Gardiner^5^ estimated the variability on a large cohort of patients, and found the following similar relationship: ln(SD) = −0.070 ⋅ Sensitivity (dB) + 2.70. Neither study, however, quantified the variability for low-sensitivity values, and assumed a similar trend for locations less than 10 and less than 15 dB, in the former and latter study, respectively. We found an analogous relationship because the variability increased with the reduction of threshold sensitivity but with a less steep slope, as expressed by the formula, ln(SD) = −0.027 ⋅ Sensitivity (dB) + 1.79. Previous studies assumed a constant increase of variability below 10 dB, but we demonstrated that it restart decreasing below 10 dB. When we ignored data below 10 dB, variability increased according to the following formula: $(\ln \left( {{\rm{SD}}} \right) = - 0.048 \cdot {\rm{Sensitivity}}\left( {{\rm{dB}}} \right) + 2.34$. The relationship between variability and threshold sensitivity is neither linear nor monotonic, and is therefore better captured by a spline function. We fitted a spline with two knots and three piecewise of regression, where each segment modeled the different behavior of variability over the dynamic range, with a variability increase between 0 and 14 dB (first segment), a reduction in variability between 15 and 32 dB (second segment), and an increase of variability between 33 and 35 dB (third segment). This polynomial function better describes the nonmonotonic relationship between variability and sensitivity.

Computer simulation may be used to compare different methods to detect perimetric progression, and consists of the simulation of longitudinal VF series with predetermined rates and patterns of progression.^39^ The previous formula by Henson et al.^3^ has been widely employed to add variability to the simulated sequences, but it is limited by a small sample size, with no measured values below 10 dB. In addition to providing a new descriptive mathematic relationship, we also report the exact amount of variability for each threshold sensitivity value (Supplementary Table S1), and these data can be used to simulate VF sequences more realistically. Because residuals do not follow a normal distribution in the low sensitivity range due to floor effect, the computation of the noise in a Gaussian form, which is based on the SD values, may be inaccurate. In this regard, recent studies^40^,^41^ have used the actual distribution of the residuals, empirically calculated on large cohort of patients with the same methodology employed in the present study. These methods, however, are difficult to replicate because raw data has not been shared in detail. In this article (see datasets in Supplementary Files S1–S3), we share with the readers the residuals, fitted, and observed values estimated with the three different models (exponential, linear, and logistic) for every eye, location, and time point. These values may be used to generate a decibel-by-decibel distribution of the residuals to replicate the aforementioned methods.

It is commonly believed that variability increases with eccentricity. Very few studies, however, quantified the effect of test location on the relationship between variability and sensitivity. Almost 35 years ago, Flammer et al.^42^ examined patients with glaucoma, glaucoma suspects, and healthy subjects, and found that fluctuation was not related to the test location in the glaucoma suspects and healthy subjects, but was slightly increased in glaucoma patients in the upper hemifield, which is the most frequently affected area. Werner et al.^28^ evaluated 67 glaucoma patients, and found that fluctuation increased with eccentricity, but the effect of test location disappeared with the correction for differences in sensitivity. These results were replicated by Boeglin and colleagues^29^ in 93 clinically stable eyes of 67 glaucoma patients. In a group of healthy subjects, Heijl et al.^12^ found contradictory results with increased fluctuation in the peripheral locations. In a subsequent study, Heijl and colleagues^33^ evaluated a small cohort of glaucoma patients with a test–retest strategy, and found that an increase of variability with eccentricity is seen in patients with mild-to-moderate damage, but the difference was no longer detected for locations with worse glaucomatous damage, defined as total deviation values worse than −10 dB. In a recent study, Gardiner^5^ tested the impact of eccentricity on variability through linear regression of large-scale longitudinal data and found an increased variability at the peripheral test locations only for sensitivity values above 28 dB. Surprisingly, we observed an increase of fluctuation related to eccentricity, as the variability was higher within 10° from fixation compared with more than 20°. Although these results are similar with those by Gardiner,^5^ the explanation of these results is not straightforward. Differences in study design, sample size, test strategy, and degrees of VF test can justify discrepancies in the study by Heijl et al.^12^ According to the hill of vision, central locations have normally higher threshold sensitivity values compared with peripheral locations, and the same sensitivity value may represent a more significant level of damage in the central area because sensitivity at central locations is normally higher than at peripheral locations. The results did not change when we repeated the analysis on the total deviation map, which accounts for deviation from normal age-matched values for each test location.

The first part of our study corroborated the familiar concept that VF data are heteroscedastic, because the amount of noise is not equally spread across the entire dynamic range of the instrument, but varies as a function of threshold sensitivity. However, data heteroscedasticity contradicts one of the main assumptions of the linear regression model, which is among the most popular methods to detect and measure perimetric progression.^26^ Violation of the homoscedasticity assumption did not bias the results of standard OLSR, but this model should not be considered the best linear unbiased estimator.^6^

Different approaches have been proposed to deal with data heteroscedasticity, and three of the most popular are transformation of the outcome variable, robust regression, and weighted regression.^6^ The first method relies on the application of some function to the dependent variable to reduce or minimize the unequal variance. Log transformation is a popular approach that does not preserve the linear relationship between the variables, but leads to an exponential relationship. Exponential regression works by compressing larger values more than smaller one, and, therefore, it can compensate for heteroscedasticity when the variance increases with the mean. In the specific setting of VF modeling, however, exponential regression is not able to account for heteroscedasticity because the variability decreases as the threshold sensitivity value increases. Other statistical methods that preserve the linear relationship are available (e.g., Box-Cox or signed modulus power transformations), but they have not been applied to VF modeling.^6^ Robust regression refers to a broad group of models resistant to the presence of multiple outliers, some of which can also deal with heteroscedasticity.^6^,^43^ Comparative studies did not find any advantage of robust regressions over classical OLSR in terms of both model fit and prediction, despite the theoretic advantages of the former models.^44^,^45^ Weighted linear regression is a third method to deal with heteroscedastic data, and unlike the OLSR, treats each observation differently as it assigns more or less weight to those measures with smaller or higher variance, respectively.^46^ Weighted linear regression requires the precise and correct estimation of the weights in a large sample, which has been used by a relatively small number of studies.^46^ Our calculated weights were estimated with a very large glaucomatous population.

Despite these considerations, weighted PLR did not add benefit to simple PLR or PER in terms of both model fit and prediction. The evidence that OLSR performs better than both weighted and robust linear regression^44^,^45^ may suggest that data heteroscedasticity is not an important primary issue in VF modeling. It may also suggest that ignoring data heteroscedasticity does not significantly affect the goodness-of-fit and the prediction ability of most of the currently used methods to identify and measure perimetric progression, which are based on simple linear regression.

In accordance with Chen et al.,^7^ all methods performed similarly for the data fit, and differences were statistically but not clinically significant. In contrast, prediction ability was considerably different among the methods, and this provides further evidence for the concept that goodness of fit and prediction ability do not coincide.^7^,^47^ PER was the best model to predict future sensitivity values in the medium and long term, and models tended to converge with the increase of VF number to make a prediction. We have previously demonstrated that VF loss across the entire perimetric range is best described by a nonlinear (i.e., logistic) rather than a linear function.^48^

This study carries all the limitations related to its retrospective nature. In addition, some factors potentially affecting fluctuation were ignored, such as increased variability at the edge of a scotoma and correlation between adjacent locations.^49^,^50^

In conclusion, VF variability is heteroscedastic and increases with the severity of glaucoma damage, but decreases with eccentricity. Weighted PLR, which accounts for heteroscedasticity, did not outperform other models for fitting long-term data. PER was the best model to predict future sensitivity values in the medium and long term. The better prediction ability of PER may be related to the nonlinear nature of long-term glaucomatous perimetric decay and the floor effect of perimetric measurements.

## Supplementary Material

Supplement 1

Supplement 2

Supplement 3

Supplement 4

Supplement 5

Supplement 6

Supplement 7

## References

[1] Qiu M, Wang SY, Singh K, Lin SC (2014). Association between visual field defects and quality of life in the United States. *Ophthalmology*.

[2] Russell RA, Crabb DP, Malik R, Garway-Heath DF (2012). The relationship between variability and sensitivity in large-scale longitudinal visual field data. *Invest Ophthalmol Vis Sci*.

[3] Henson DB, Chaudry S, Artes PH, Faragher EB, Ansons A (2000). Response variability in the visual field: comparison of optic neuritis, glaucoma, ocular hypertension, and normal eyes. *Invest Ophthalmol Vis Sci*.

[4] Artes PH, Iwase A, Ohno Y, Kitazawa Y, Chauhan BC (2002). Properties of perimetric threshold estimates from full threshold, SITA standard, and SITA fast strategies. *Invest Ophthalmol Vis Sci*.

[5] Gardiner SK (2018). Differences in the relation between perimetric sensitivity and variability between locations across the visual field. *Invest Ophthalmol Vis Sci*.

[6] Kaufamn RL (2013). *Heteroskedasticity in Regression: Detection and Correction*.

[7] Chen A, Nouri-Mahdavi K, Otarola FJ, Yu F, Afifi AA, Caprioli J (2014). Models of glaucomatous visual field loss. *Invest Ophthalmol Vis Sci*.

[8] Caprioli J, Mock D, Bitrian E (2012). Author response: on alternative methods for measuring visual field decay: Tobit linear regression. *Invest Ophthalmol Vis Sci*.

[9] Lee JM, Nouri-Mahdavi K, Morales E, Afifi A, Yu F, Caprioli J (2014). Comparison of regression models for serial visual field analysis. *Jpn J Ophthalmol*.

[10] Yohannan J, Wang J, Brown J (2017). Evidence-based criteria for assessment of visual field reliability. *Ophthalmology*.

[11] R Development Core Team. R: A language and enviroment for statistical computing Vienna, Austria R Foundation for Statistical Computing 2010

[12] Heijl A, Lindgren G, Olsson J (1987). Normal variability of static perimetric threshold values across the central visual field. *Arch Ophthalmol*.

[13] Spry PG, Johnson CA (2001). Senescent changes of the normal visual field: an age-old problem. *Optom Vis Sci*.

[14] Garway-Heath DF, Crabb DP, Bunce C (2015). Latanoprost for open-angle glaucoma (UKGTS): a randomised, multicentre, placebo-controlled trial. *Lancet*.

[15] Musch DC, Lichter PR, Guire KE, Standardi CL (1999). The Collaborative Initial Glaucoma Treatment Study: study design, methods, and baseline characteristics of enrolled patients. *Ophthalmology*.

[16] Leske MC, Heijl A, Hyman L, Bengtsson B (1999). Early Manifest Glaucoma Trial: design and baseline data. *Ophthalmology*.

[17] Ederer F, Gaasterland DE, Sullivan EK;, AGIS Investigators. (1994). The Advanced Glaucoma Intervention Study (AGIS): 1. Study design and methods and baseline characteristics of study patients. *Control Clin Trials*.

[18] Caprioli J (2008). The importance of rates in glaucoma. *Am J Ophthalmol*.

[19] Lee JW, Morales E, Yu F (2014). Effect of cataract extraction on the visual field decay rate in patients with glaucoma. *JAMA Ophthalmol*.

[20] Junoy Montolio FG, Wesselink C, Gordijn M, Jansonius NM (2012). Factors that influence standard automated perimetry test results in glaucoma: test reliability, technician experience, time of day, and season. *Invest Ophthalmol Vis Sci*.

[21] Mutlukan E (1994). The effect of refractive blur on the detection sensitivity to light offsets in the central visual field. *Acta Ophthalmol (Copenh)*.

[22] Kutzko KE, Brito CF, Wall M (2000). Effect of instructions on conventional automated perimetry. *Invest Ophthalmol Vis Sci*.

[23] Gracitelli CPB, Zangwill LM, Diniz-Filho A (2018). Detection of glaucoma progression in individuals of African descent compared with those of European descent. *JAMA Ophthalmol*.

[24] Diniz-Filho A, Delano-Wood L, Daga FB, Cronemberger S, Medeiros FA (2017). Association between neurocognitive decline and visual field variability in glaucoma. *JAMA Ophthalmol*.

[25] Blumenthal EZ, Sample PA, Berry CC (2003). Evaluating several sources of variability for standard and SWAP visual fields in glaucoma patients, suspects, and normals. *Ophthalmology*.

[26] Spry PG, Johnson CA (2002). Identification of progressive glaucomatous visual field loss. *Surv Ophthalmol*.

[27] Zulauf M, Caprioli J, Hoffman DC, Tressler CS, Mills RP, Heijl A (1991). Fluctuation of the differential light sensitivity in clinically stable glaucoma patients. Amsterdam: Kugler Publications.

[28] Werner EB, Ganiban G, Balaszi AG, Mills RP, Heijl A (1991). Effect of test point location on the magnitude of threshold fluctuation in glaucoma patients undergoing automated perimetry. Amsterdam: Kugler Publications.

[29] Boeglin RJ, Caprioli J, Zulauf M (1992). Long-term fluctuation of the visual field in glaucoma. *Am J Ophthalmol*.

[30] Chauhan BC, Tompkins JD, LeBlanc RP, McCormick TA (1993). Characteristics of frequency-of-seeing curves in normal subjects, patients with suspected glaucoma, and patients with glaucoma. *Invest Ophthalmol Vis Sci*.

[31] Spry PG, Johnson CA, McKendrick AM, Turpin A (2001). Variability components of standard automated perimetry and frequency-doubling technology perimetry. *Invest Ophthalmol Vis Sci*.

[32] Chauhan BC, Johnson CA (1999). Test-retest variability of frequency-doubling perimetry and conventional perimetry in glaucoma patients and normal subjects. *Invest Ophthalmol Vis Sci*.

[33] Heijl A, Lindgren A, Lindgren G (1989). Test-retest variability in glaucomatous visual fields. *Am J Ophthalmol*.

[34] Piltz JR, Starita RJ (1990). Test-retest variability in glaucomatous visual fields. *Am J Ophthalmol*.

[35] Gardiner SK, Demirel S, Johnson CA (2008). Is there evidence for continued learning over multiple years in perimetry?. *Optom Vis Sci*.

[36] Gardiner SK, Crabb DP (2002). Examination of different pointwise linear regression methods for determining visual field progression. *Invest Ophthalmol Vis Sci*.

[37] Gardiner SK, Crabb DP (2002). Frequency of testing for detecting visual field progression. *Br J Ophthalmol*.

[38] Turpin A, McKendrick AM (2011). What reduction in standard automated perimetry variability would improve the detection of visual field progression?. *Invest Ophthalmol Vis Sci*.

[39] Spry PG, Bates AB, Johnson CA, Chauhan BC (2000). Simulation of longitudinal threshold visual field data. *Invest Ophthalmol Vis Sci*.

[40] Russell RA, Garway-Heath DF, Crabb DP (2013). New insights into measurement variability in glaucomatous visual fields from computer modelling. *PLoS One*.

[41] Wu Z, Medeiros FA (2018). Development of a visual field simulation model of longitudinal point-wise sensitivity changes from a clinical glaucoma cohort. *Transl Vis Sci Technol*.

[42] Flammer J, Drance SM, Fankhauser F, Augustiny L (1984). Differential light threshold in automated static perimetry. Factors influencing short-term fluctuation. *Arch Ophthalmol*.

[43] Atkinson AC, Riani M, Torti F (2016). Robust methods for heteroskedastic regression. *Comp Stat Data Analysis*.

[44] Bryan SR, Vermeer KA, Eilers PH, Lemij HG, Lesaffre EM (2013). Robust and censored modeling and prediction of progression in glaucomatous visual fields. *Invest Ophthalmol Vis Sci*.

[45] Fujino Y, Murata H, Mayama C, Asaoka R (2015). Applying “Lasso'' regression to predict future visual field progression in glaucoma patients. *Invest Ophthalmol Vis Sci*.

[46] Carroll RJ, Ruppert D (1988). *Transformation and Weighting in Regression*.

[47] McNaught AI, Crabb DP, Fitzke FW, Hitchings RA (1995). Modelling series of visual fields to detect progression in normal-tension glaucoma. *Graefes Arch Clin Exp Ophthalmol*.

[48] Otarola F, Chen A, Morales E, Yu F, Afifi A, Caprioli J (2016). Course of glaucomatous visual field loss across the entire perimetric range. *JAMA Ophthalmol*.

[49] Haefliger IO, Flammer J (1991). Fluctuation of the differential light threshold at the border of absolute scotomas. Comparison between glaucomatous visual field defects and blind spots. *Ophthalmology*.

[50] Lachenmayr BJ, Kiermeir U, Kojetinsky S (1995). Points of a normal visual field are not statistically independent. *Ger J Ophthalmol*.

