# Supplementary material for: Quantification of Visual Field Variability in Glaucoma: Implications for Visual Field Prediction and Modeling
Source: Transl Vis Sci Technol. 2019 Oct 17;8(5):25. doi: 10.1167/tvst.8.5.25 (PMC6798312; doi:10.1167/tvst.8.5.25)
Supplement: Supplement 1 [file tvst-08-05-19_s01.docx]

| **Supplementary Table 1.** Standard deviation of the residuals for each observed sensitivity value calculated with the pointwise exponential, linear, and logistic models. | | | |
| --- | --- | --- | --- |
| **Sensitivity (dB)** | **EXPONENTIAL** | **LINEAR** | **LOGISTIC** |
| **35** | 2.31 | 2.19 | 2.24 |
| **34** | 2.12 | 1.89 | 1.85 |
| **33** | 2.03 | 1.68 | 1.67 |
| **32** | 2.08 | 1.62 | 1.62 |
| **31** | 2.11 | 1.61 | 1.61 |
| **30** | 2.23 | 1.69 | 1.68 |
| **29** | 2.32 | 1.78 | 1.75 |
| **28** | 2.43 | 1.86 | 1.81 |
| **27** | 2.61 | 2.02 | 1.94 |
| **26** | 2.84 | 2.19 | 2.09 |
| **25** | 3.05 | 2.36 | 2.24 |
| **24** | 3.29 | 2.57 | 2.43 |
| **23** | 3.53 | 2.76 | 2.61 |
| **22** | 3.77 | 2.97 | 2.78 |
| **21** | 4.03 | 3.19 | 2.99 |
| **20** | 4.31 | 3.42 | 3.21 |
| **19** | 4.51 | 3.62 | 3.42 |
| **18** | 4.75 | 3.84 | 3.64 |
| **17** | 5.00 | 4.09 | 3.93 |
| **16** | 5.12 | 4.25 | 4.07 |
| **15** | 5.31 | 4.47 | 4.29 |
| **14** | 5.41 | 4.63 | 4.49 |
| **13** | 5.47 | 4.76 | 4.63 |
| **12** | 5.50 | 4.89 | 4.79 |
| **11** | 5.52 | 4.99 | 4.93 |
| **10** | 5.44 | 5.02 | 5.00 |
| **9** | 5.40 | 5.11 | 5.10 |
| **8** | 5.31 | 5.17 | 5.20 |
| **7** | 5.16 | 5.10 | 5.18 |
| **6** | 4.95 | 5.04 | 5.17 |
| **5** | 4.88 | 5.12 | 5.28 |
| **4** | 4.74 | 5.15 | 5.32 |
| **3** | 4.56 | 5.04 | 5.30 |
| **2** | 4.18 | 4.91 | 5.16 |
| **1** | 4.06 | 4.92 | 5.25 |
| **0** | 3.36 | 4.23 | 4.61 |
| dB: decibel | | | |
