# Supplementary figures and images for: Quantification of Visual Field Variability in Glaucoma: Implications for Visual Field Prediction and Modeling
Source: Transl Vis Sci Technol. 2019 Oct 17;8(5):25. doi: 10.1167/tvst.8.5.25 (PMC6798312; doi:10.1167/tvst.8.5.25)

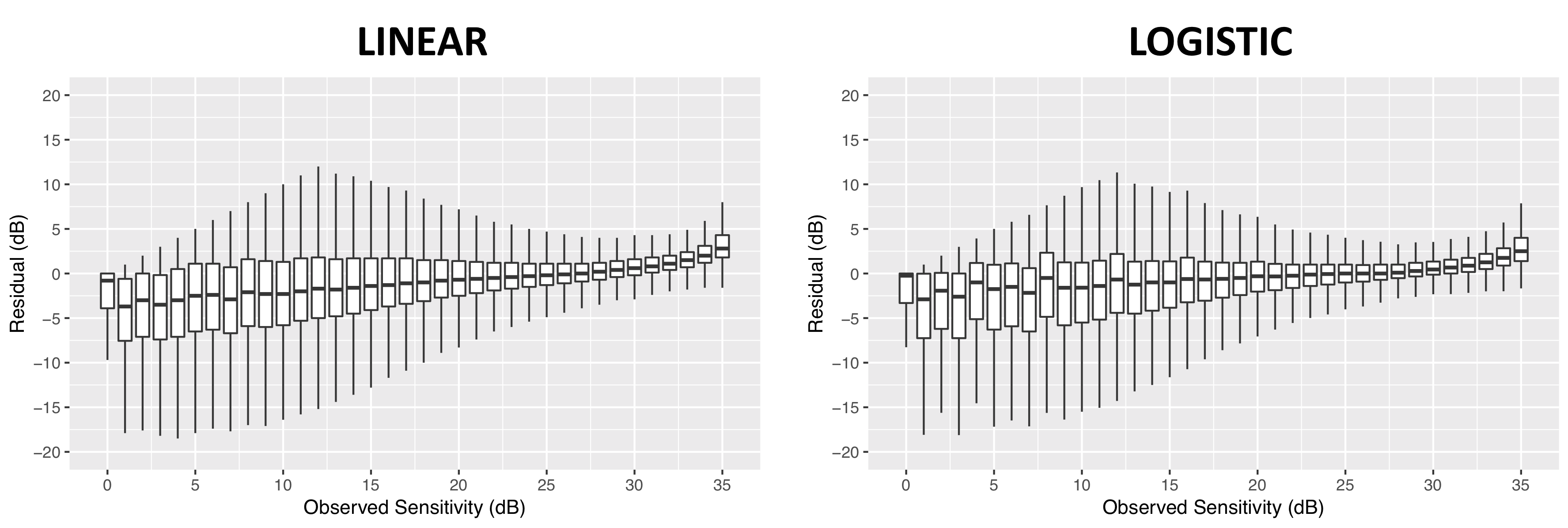

Supplement: Supplement 2 [file tvst-08-05-19_s02.tif]

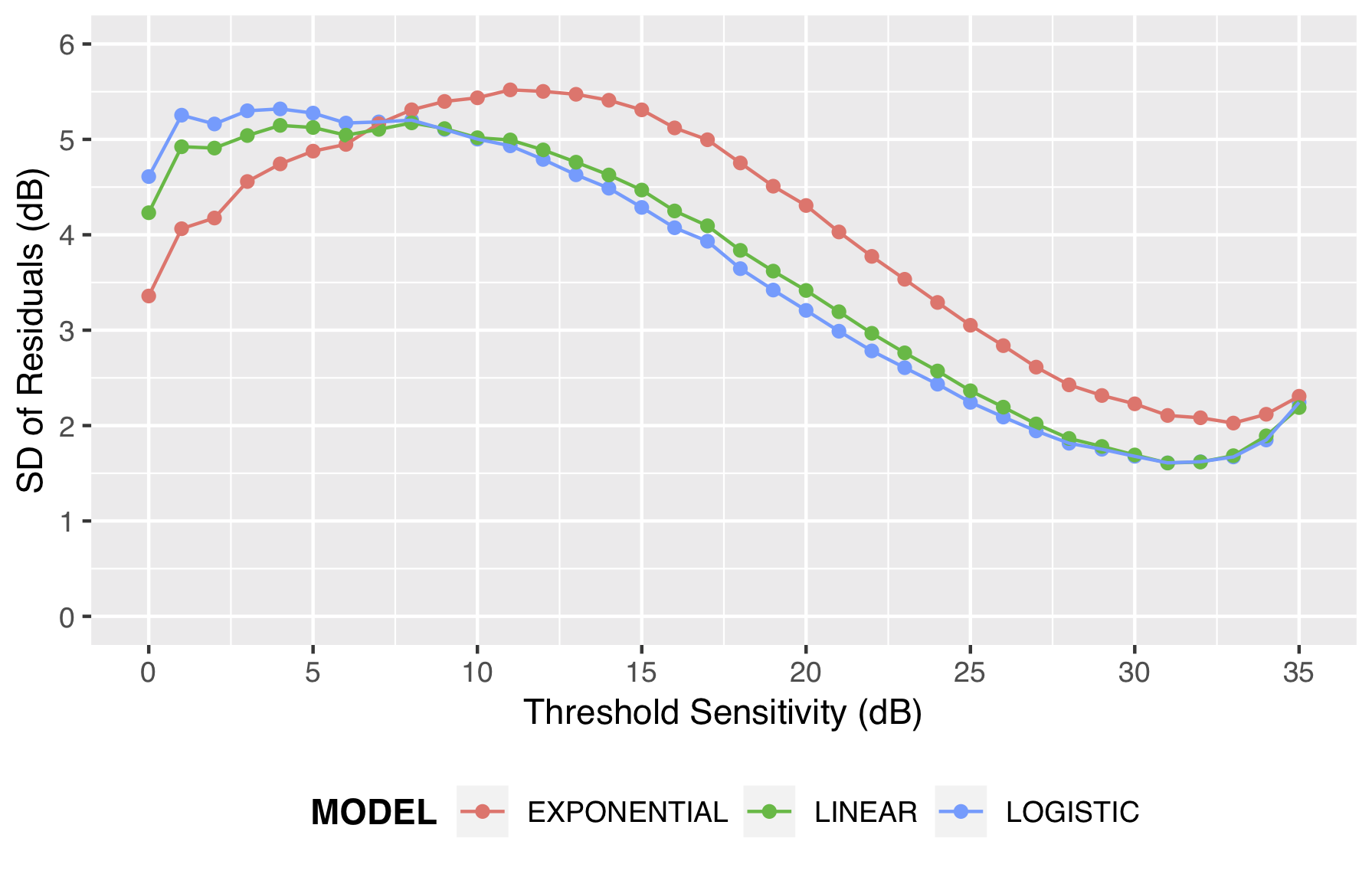

Supplement: Supplement 3 [file tvst-08-05-19_s03.tif]

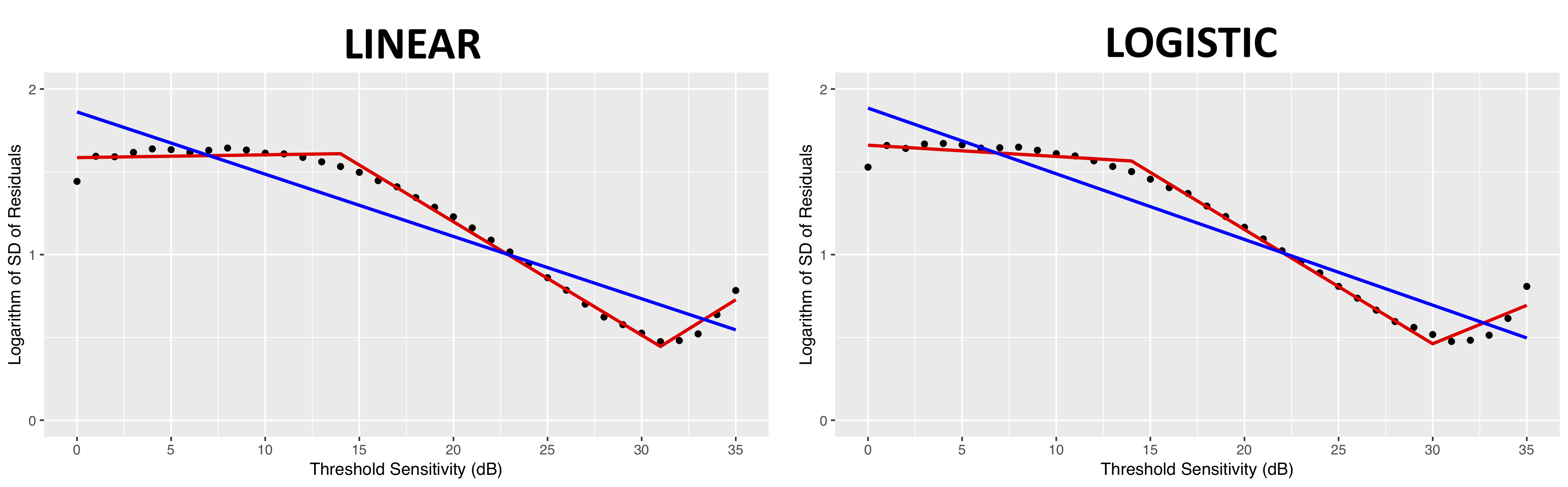

Supplement: Supplement 4 [file tvst-08-05-19_s04.tif]

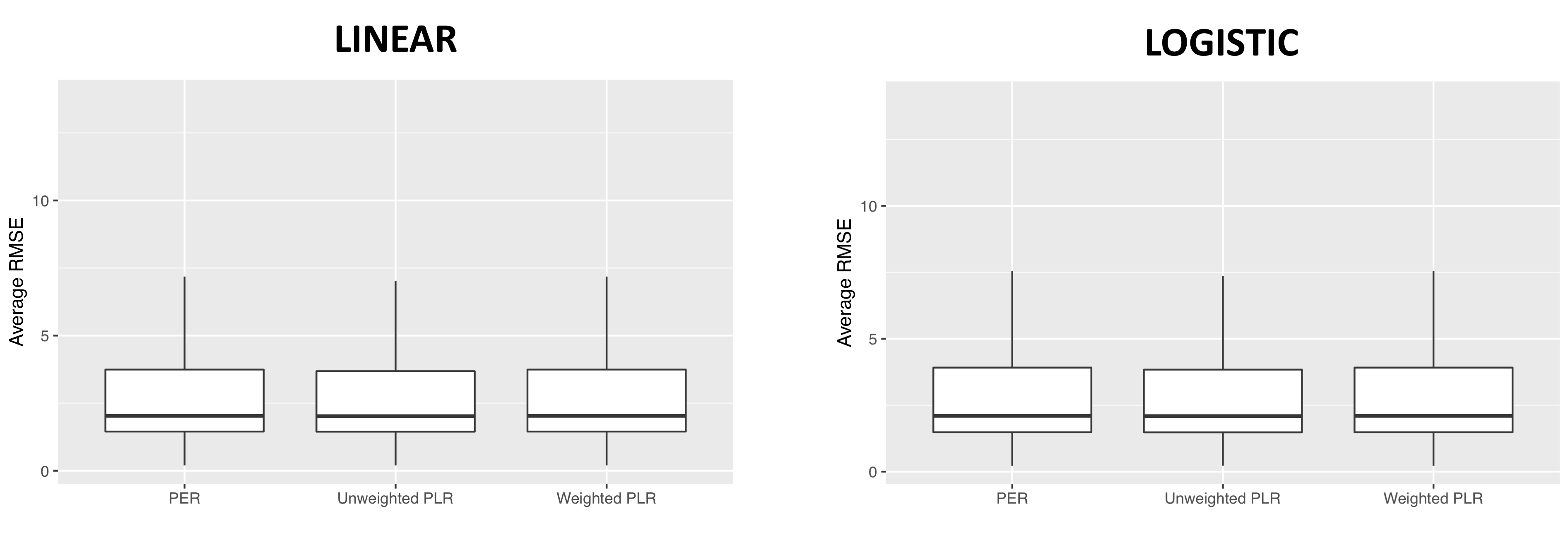

Supplement: Supplement 5 [file tvst-08-05-19_s05.tif]

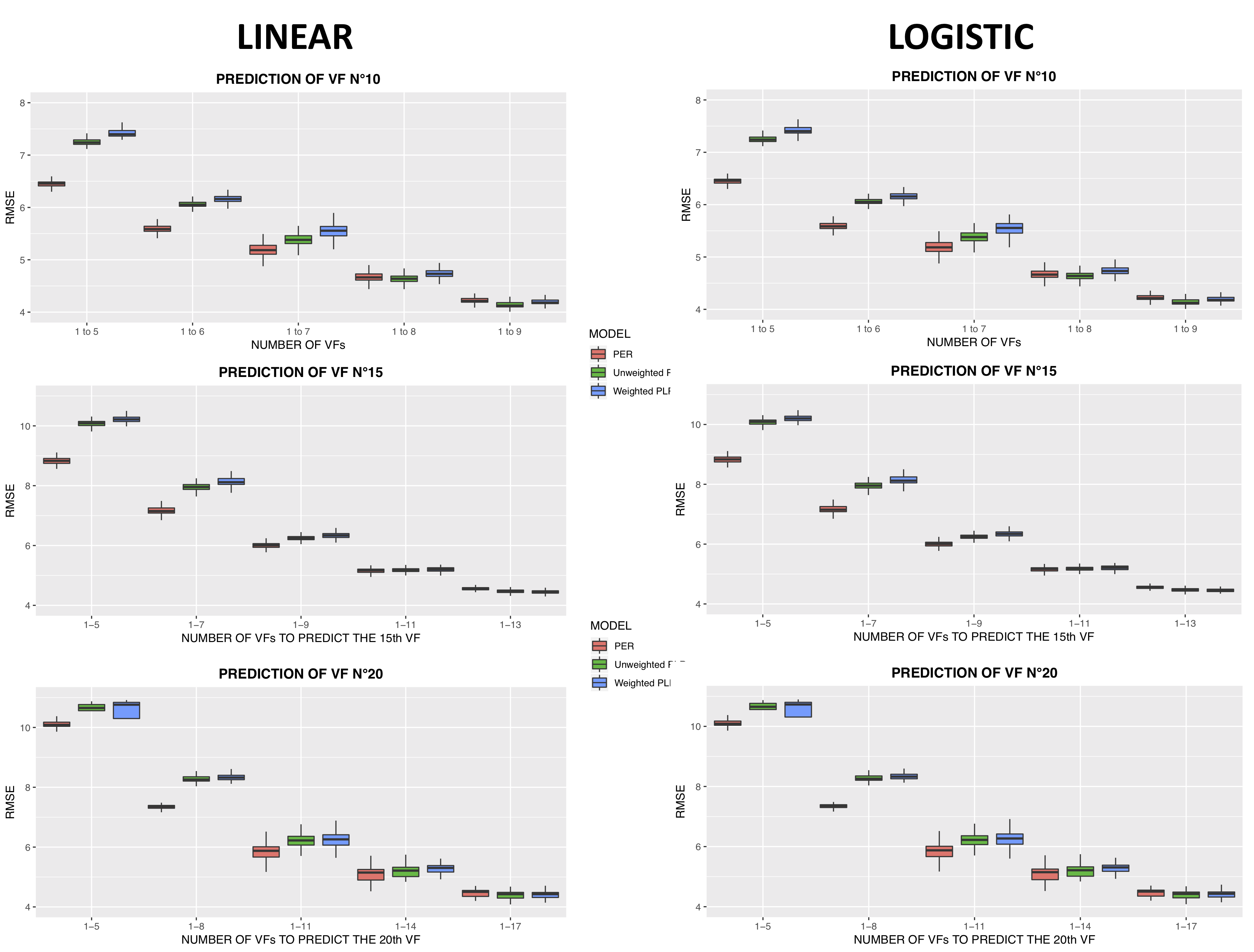

Supplement: Supplement 6 [file tvst-08-05-19_s06.tif]
